# Correction: Mitochondrial impairment, decreased Sirtuin activity and protein acetylation in dorsal root ganglia in Friedreich ataxia models

**DOI:** 10.1007/s00018-025-05948-7

**Published:** 2025-11-26

**Authors:** Arabela Sanz-Alcázar, Elena Britti, Fabien Delaspre, Marta Medina-Carbonero, Maria Pazos-Gil, Jordi Tamarit, Joaquim Ros, Elisa Cabiscol

**Affiliations:** Departament de Ciències Mèdiques Bàsiques, Facultat de Medicina, Universitat de Lleida, IRBLleida, Edifici Biomedicina I, Av. Rovira Roure, 80, 25198 Lleida, Catalonia Spain

**Correction: Cellular and Molecular Life Sciences (2023) 81:12**

10.1007/s00018-023-05064-4

In this article the statement in the Funding information section was incorrectly given as

'Open Access funding provided thanks to the CRUE-CSIC agreement with Springer Nature. This work was supported by Ministerio de Economía y Competitividad, MINECO (Spain) [grants PN-P21018 and PDC-N21019] and Generalitat de Catalunya [SGR2009-00196]. ASA received a Ph.D. fellowship from the Generalitat de Catalunya. Maria Pazos received a PhD fellowship from the Universitat de Lleida.'

and should have read

'Open Access funding provided thanks to the CRUE-CSIC agreement with Springer Nature. This work was supported by: 1) Project PID2020-118296RB-I00 funded by MICIU/AEI/10.13039/501100011033; 2) Project CPP2021-008554 funded by MICIU/AEI/10.13039/501100011033 and by the European Union NextGenerationEU/ PRTR; 3) Project PDC2021-120758-I00 funded by MICIU/AEI /10.13039/501100011033 and by the European Union NextGenerationEU/ PRTR; 4) Project 2021-SGR 00323 funded by Generalitat de Catalunya. ASA received a Ph.D. fellowship from the Generalitat de Catalunya. Maria Pazos received a PhD fellowship from the Universitat de Lleida.'.

The original article has been corrected.

